# Effect of long-term tillage on soil aggregates and aggregate-associated carbon in black soil of Northeast China

**DOI:** 10.1371/journal.pone.0199523

**Published:** 2018-06-28

**Authors:** Hongbing Zheng, Wuren Liu, Jinyu Zheng, Yang Luo, Ruiping Li, Hao Wang, Hua Qi

**Affiliations:** 1 Agronomy College, Agricultural University of Shenyang, Shenyang, China; 2 Research Institute of Agricultural Resources and Environment, Jilin Academy of Agricultural Science, Changchun, China; 3 Key Laboratory of Crop Ecophysiology and Farming System in Northeast China, Ministry of Agriculture, Changchun, China; Shandong University, CHINA

## Abstract

Soil tillage can affect the stability and formation of soil aggregates by disrupting soil structure. Frequent tillage deteriorates soil structure and weakens soil aggregates, causing them to be susceptible to decay. Different types of tillage systems affect soil physical properties and organic matter content, in turn influencing the formation of aggregates. The objective of this study was to evaluate the effect of long-term tillage on soil aggregates and aggregate-associated carbon in a black soil of Northeast China and to identify the optimal conservation tillage in this system. This research was conducted on a long-term tillage experimental field established in 1983 at the Jilin Academy of Agricultural Sciences, Gongzhuling, China. Plots were treated with four tillage systems including no tillage (NT), spacing tillage (ST), moldboard plowing (MP), and conventional tillage (CT). We took samples every 10cm from 0-60cm depth and demonstrated that water-stable soil aggregates >0.25mm in diameter accounted for over 66.0% of total aggregates for all tillage treatments, and the percentage for the ST treatment was 34.5% higher than in the other treatments. The NT treatment had the highest effect at 0–10cm depth, while the effect for the ST treatment was highest at 0–30cm. SOC storage decreased with soil depth, with a significant accumulation at 0-20cm depth. Across treatments, aggregate-associated C at a depth of 0–10cm was higher in the NT and ST treatments than in the MP and CT treatments. The advantage of the NT treatment weakened with soil depth, while the amount of aggregate-associated C remained higher for the ST treatment. There were more macro-aggregates in the ST and NT treatments than in the MP and CT treatments, while the MP and CT treatments had more micro-aggregates. The sum of macro-aggregate contributing rates for soil organic C (SOC) was significantly superior to that of the micro-aggregates. Water-stable aggregates increased by 34.5% in the ST treatment, effectively improving the soil structure. Furthermore, 0.25–1.00 and 1–2mm aggregates had the highest SOC storage and responded rapidly to the various tillage treatments. Hence, they can serve as indicators for the long-term influence of different tillage treatments on the distribution of aggregates and SOC.

## Introduction

Soil is considered the ‘skin’ of the earth [1], with soil organic carbon (SOC) as the protein that protects the ‘skin’ [2]. SOC is a key indicator of soil quality [3], is the basis of soil fertility and function [4–5], and is important for cementing substances as part of the formation of soil aggregates. SOC affects the number and distribution of differently sized soil aggregates [6]. Soil aggregates are the basic ‘cells’ of the soil structure and play an important role in improving soil carbon sequestration and fertility [7]. Stable soil aggregates not only reduce soil erosion-induced SOC loss, but also inhibit microbial and enzymatic decomposition of SOC through coating and isolation effects [8–9]. Physical fraction is widely used to study the storage and turnover of soil organic matter (SOC), because it incorporates three levels of analysis by examining three sizes of aggregate. Previous studies have demonstrated that the interaction between soil structure and aggregates determines the quality of the SOC pool. SOC is primarily distributed in water-stable aggregates of larger sizes (> 1mm) and SOC content increases with aggregate diameter [10–11]. The combined application of chemical fertilizer and straw greatly improves SOC accumulation in water-stable aggregates of this size [12–13].

Tilling can play an important role in increasing crop yield, thereby improving food security worldwide by making crop growth more successful and controlling competition by weeds [14]. However, many studies have demonstrated that intensive tillage deteriorates soil structure and enhance soil erosion [15]. Specially, moldboard plowing may damage the pore continuity and aggregate stability resulting in sediment mobilization, erosion, and surface hardening [16]. This effect frequently exposes aggregates to physical disruption [17]. The resulting breaking of aggregates enhances the accessibility of organic matter (OM) to microorganisms, stimulating oxidation and loss of organic matter [18]. Declines in organic matter are thus usually accompanied by a decrease in the number of water-stable aggregates [19]. Intensive tillage that accelerates the conversion of soil macro-aggregates is the main cause of SOC loss [20]. However, no-tillage practices have been adopted worldwide because they are perceived to be an efficient technology for soil conservation and sustainable agriculture in developed countries such as the USA, Brazil, Canada, Chile, Paraguay, and Australia [21]. Under no tillage, crop residue decomposes at a slower rate, leading to a gradual build-up and increase in soil organic carbon (SOC). The resulting substrate from residue decomposition contributes to stabilizing soil aggregates [22–23]. In addition, some previous studies showed that no tillage with straw return improved the SOC content in differently sized aggregates at all soil depths, but showed a minimal influence on SOC content and on oxidative stability, which primarily depend on 0.05–0.25mm micro-aggregates [24].

Black soil in the Northeast China Plain is an inherently productive and fertile soil resource [24]. However, soil organic matter has declined because of long-term intensive cultivation practices and the amount of soil organic matter has severely deteriorated [15]. Adoption of appropriate tillage systems and cropping regimes is crucial for increasing soil organic matter and enhancing the stability of aggregates [25]. In the past decades, some studies on the distribution of water-stable aggregates and SOC in Mollisols in Northeast China focused on different plowing layers and topography [26]. Few studies have shed light on the soil aggregate content variation and on distribution characteristics of aggregate-associated C under long-term conservation tillage, which incorporates the previous year’s crop residue during the subsequent planting [27–28]. The effect of long-term conservative tillage on the aggregate size and distribution characteristics of aggregate-associated C remains especially unclear in the black soil of Northeast China. In this study, we hypothesized that intensive tillage deteriorates soil structure and reduces soil aggregates and SOC concentration, meanwhile increases the rate of aggregate decay. To this end, we measured the content of water-stable soil aggregates and aggregate-associated C to investigate water-stable aggregates and SOC distribution characteristics with soil depth under long-term spacing tillage (ST), no tillage (NT), moldboard plowing (MP), and conventional tillage (CT) treatments. We determined the extent of difference in aggregate and aggregate-associated C among different tillage treatments in the black soil of Northeast China. Our objective in the present study was to quantify the impact of long-term tillage systems on soil aggregates, aggregate-associated carbon and stability (*GMD*, *MWD*, and *E*_*LT*_) of soil aggregates and to identify the optimal conservative tillage systems for sustainable agriculture.

## Materials and methods

### Experimental site

**Fig 1:**
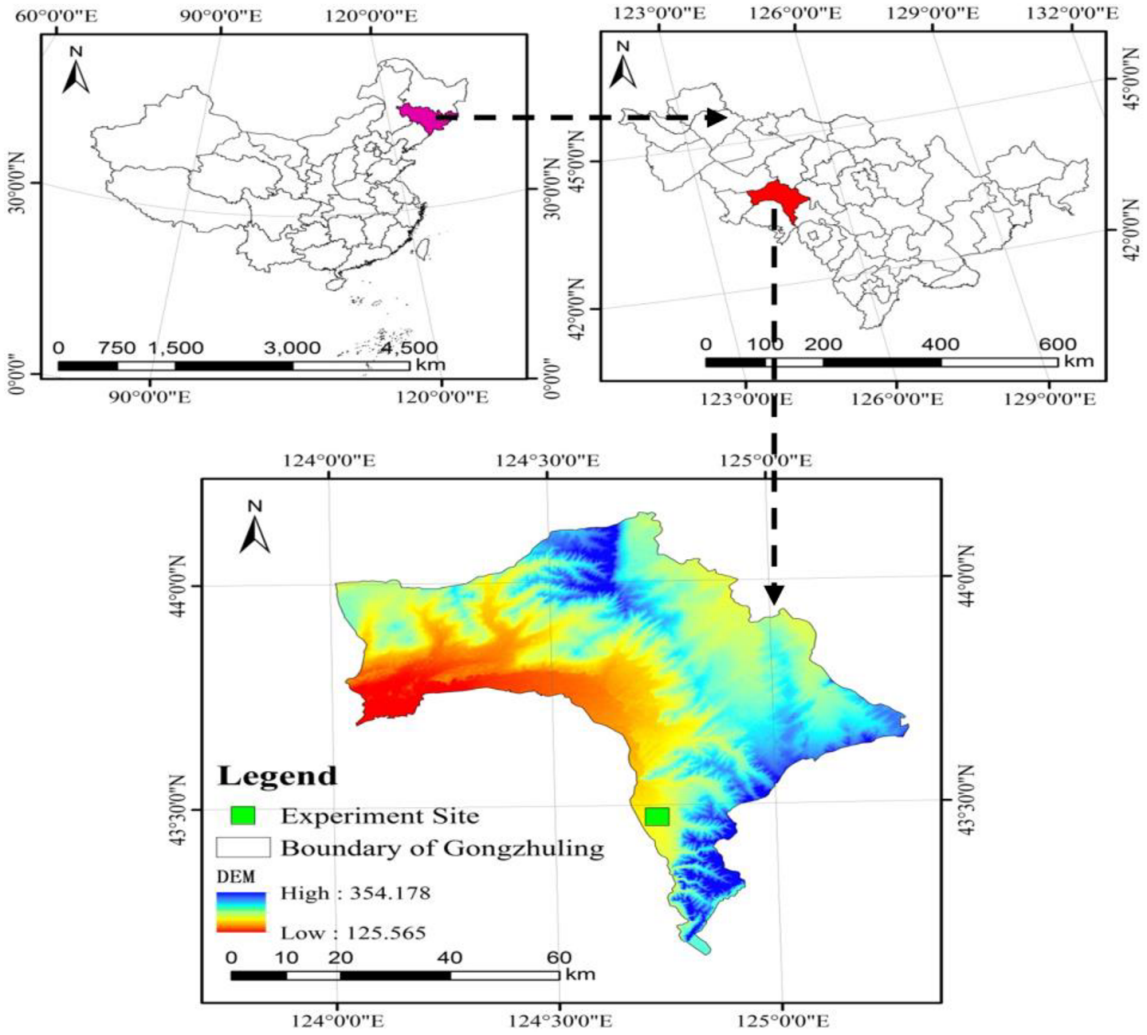
Location of the long term experimental plot.

**Fig 2:**
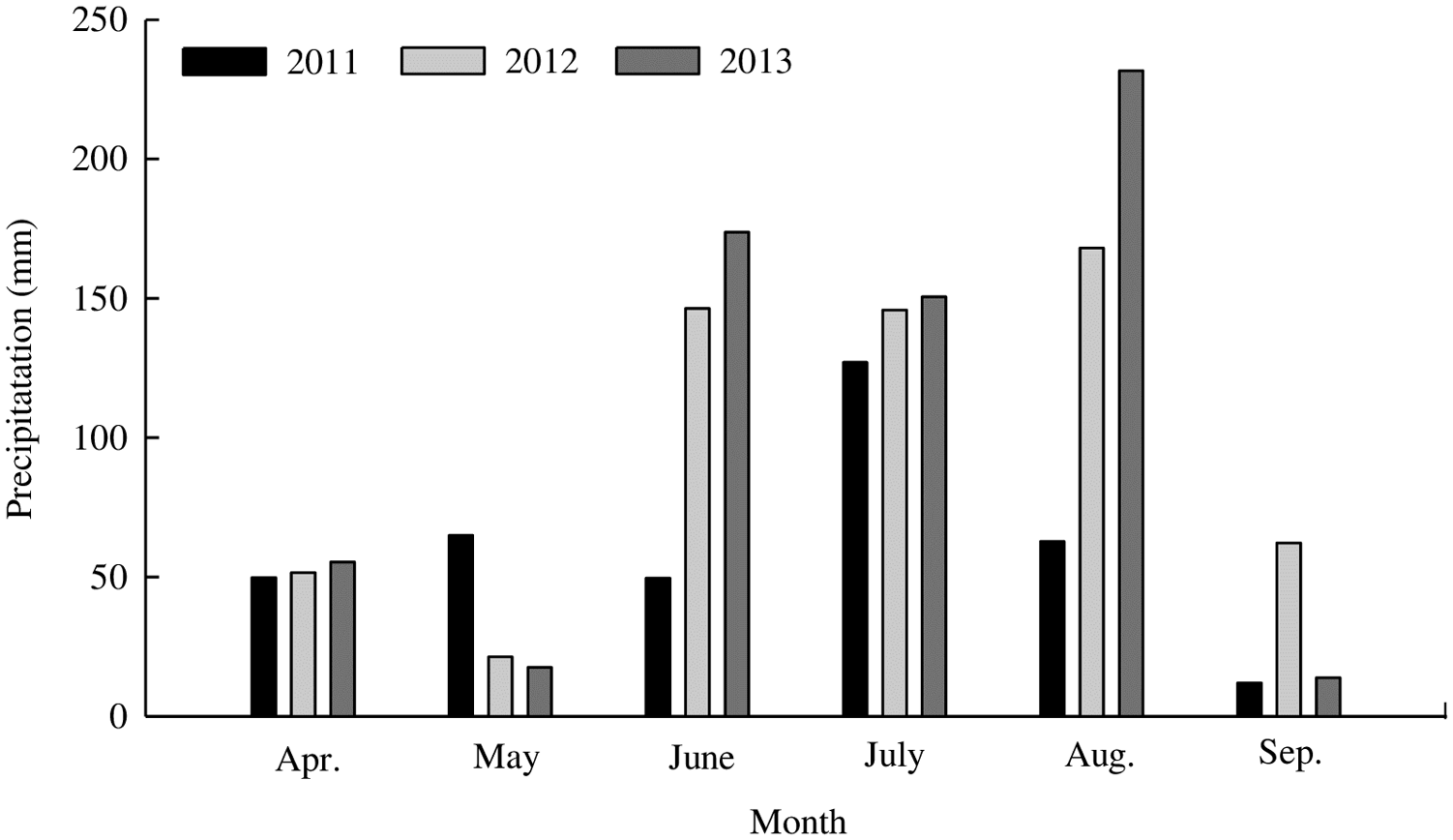
Precipitation during each month of the rainy season for 2011–2013 at the experimental site.

The research area was an experimental field at the Jilin Academy of Agricultural Sciences that had the rights of soil land use and permitted to this study, Gongzhuling, Jinlin Province, China (43°45′N, 125°01′E) (Fig 1), which experiences a mid-temperate continental monsoon climate (annual average temperature 4.5°C; annual precipitation 567mm, concentrated in June–August; Fig 2). The soil is classified as black soil (Typic Hapludoll, USDA Soil Taxonomy) with a clay loam texture (the average soil texture was 36.0% clay, 24.5% silt, and 39.5% sand). The initial soil organic C in the 0-20cm layer at the start of the experiment was 13.2g kg^-1^. The mass fractions of total nitrogen (N), phosphorus (P), and potassium (K) were 0.15, 0.05, and 2.26%, respectively, and densities of alkali-hydrolysable N, rapidly available P, and available K were 146.36, 13.50, and 152.32 mg kg^-1^, respectively. The pH and cation exchange capacity (CEC) of the topsoil (0-20cm) was 6.5 and 28.2cmol/kg. Chemical properties were measured according to standard methods, as described by Wen *et al*. [29] and the values of soil indicators were repeated ten times.

### Experimental design

**Fig 3:**
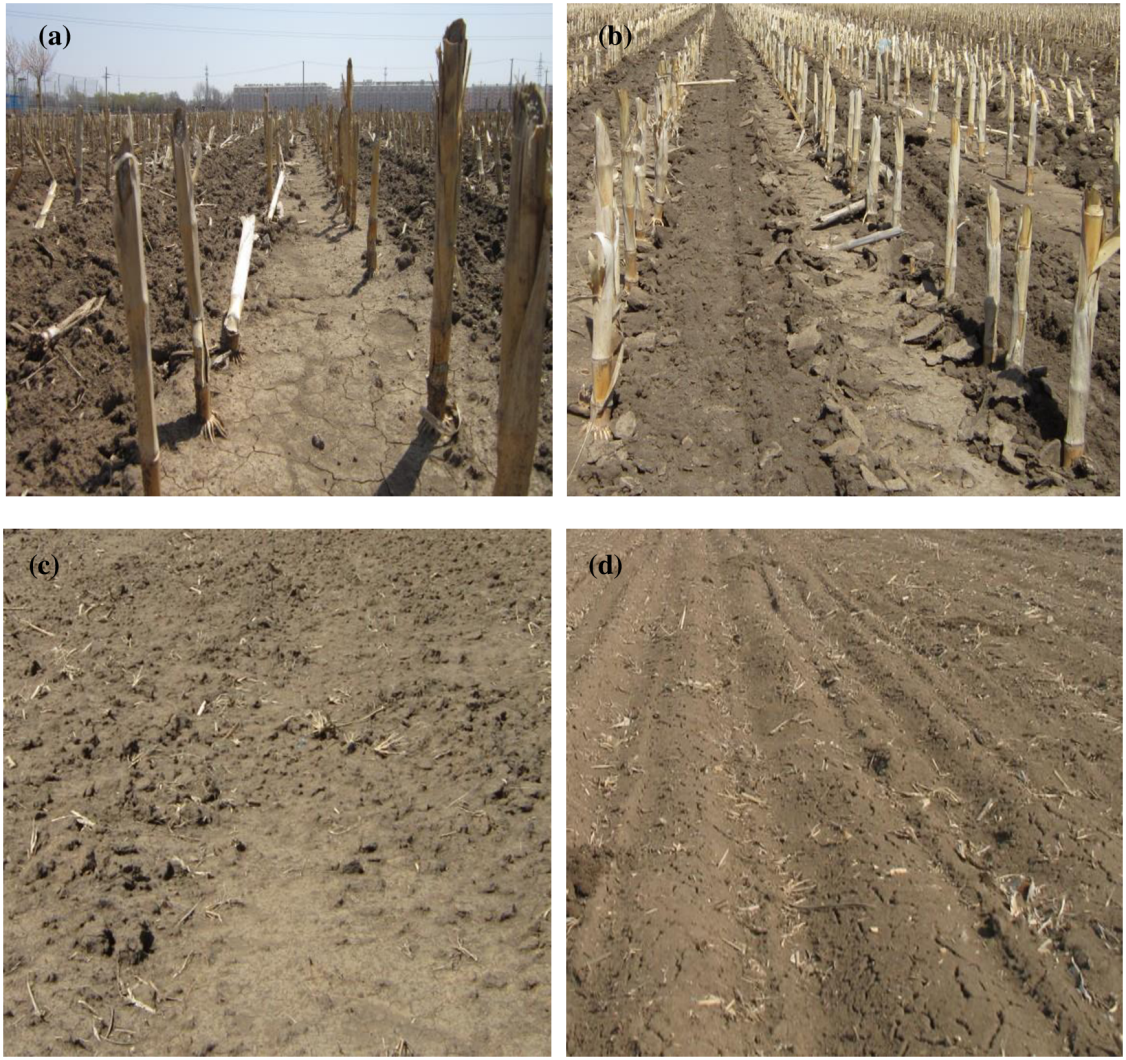
Photographs of tillage treatments. Spacing tillage (a), no-tillage (b), moldboard tillage (c), and conventional tillage (d) in the experimental plots (2013).

The experiment began in 1983 and involved four methods of tillage—spacing tillage (Fig 3a), no-tillage (Fig 3b), moldboard tillage (Fig 3c), and conventional tillage (Fig 3d) as the control—in a randomized block design with three replications (plots) per treatment, with each plot measuring 1200m^2^ (150m × 8m). The total area of the test sites was 15000m^2^. Maize (*Zea mays* ‘Zhengdan 958’) was grown each year at a density of 60000 plants ha^-1^, with line spacing of 65cm and row spacing of 25cm. The fertilizer dose comprised 243 N kg ha^-1^, 92 kg ha^-1^ P_2_O_5_, and 80 kg ha^-1^ K_2_O. Seeds were sown annually on 1 May and the crop harvested on 1 October. Field operations and the relevant details of each method of tillage are shown in Table 1.

**Table 1:** Field operations and other details of the four methods of tillage.

Methods	Details of field operations
Spacing tillage (ST)	A new conservative tillage system widely adopted by farmers in Northeast China because of the advantages of no-tillage and vertical tillage. Modifies the conventional tillage method (with ridge distance of 65cm) to include a 90cm wide sub-soiling belt and a 40cm strip for the crop. 40-45cm height of corn stubble was retained after harvesting to avoid soil erosion. Corn seeds with base fertilizer were planted in soil by a no-till planter (2BMZF-6) in the spring each year. The herbicides (acetochlor and atrazine) were sprinkled by a spraying machine (DYJ160) after seeding. Top-dressing and sub-soiling with 25-30cm depth were done with a deep tillage machine (3ZSF-6) in the jointing stage of corn (8 leaves with collars). The straws were removed from the field except for the high stubble after corn harvest in autumn.
No tillage (NT)	Planting with base fertilizer was done by no-till planter (2BMZF-4) that cuts the corn residue in spring. The herbicides (acetochlor and atrazine) were sprinkled by a spraying machine (DYJ160) after seeding. The straws were removed except for 40-45cm high stubble for conserving soil after corn harvest in autumn.
Moldboard plowing (MP)	A conventional planter (2BDJ-3) was used for sowing and base fertilizer in the spring each year. The seeding zone was compacted by a compacting machine (1YMZ-6). The herbicides (acetochlor and atrazine) were sprinkled by a corn spraying machine (DYJ160) after seeding. Top-dressing and inter tillage were carried out when the corn was at the jointing stage (8 leaves with collars). Straws, except for roots, were removed from the field and soil was turned to a depth of 20 to 25cm by using a turnover plow (1LFT-535) after harvest in autumn.
Conventional tillage (CT)	Conventional tillage commonly used by farmers in China that differs from moldboard plowing. Sowing and base fertilizer were by a conventional planter (2BDJ-3) in the spring. The herbicides (acetochlor and atrazine) were sprinkled by a spraying machine (DYJ160) after seeding. Top-dressing and inter tillage were carried out when the corn was at jointing stage (8 leaves with collars). Straws, except for roots, were removed from the field and soil was tilled to a depth of 8 to 10cm by using a rotary cultivator for seed preparation after harvest in the autumn.

### Sampling methods

Soil samples were collected by a soil sampler (diameter of 8cm) at six depths (0–10, 10–20, 20–30, 30–40, 40–50, and 50–60cm) in October 2013, after the autumn harvest. What is more, our study did not involve endangered or protected species. In addition, the sampling sites were distributed in the ridge in an ‘S’ shape. The sampling at each depth was repeated seven times, and these were mixed to create a representative sample. Soil samples were kept and transported to the laboratory in aluminum boxes to avoid destroying the aggregates. Field-moist soils were dried until they reached a gravimetric water content of about 100g H_2_O kg^-1^, when large soil blocks were separated from the fragile zone manually, sieved using a 25mm sieve to remove grass and roots, and dried at ambient temperature.

### Testing for water-stable aggregates

50g of dried soil from each sample was subjected to a set of sieves (with screen mesh: 2, 1, 0.25, 0.053, and 0.002mm), which was immersed in water and soaked for 10min to prevent breaking aggregates. After soaking, while still submerged, the sieve was shaken for 2min using aggregate wet screen (TTF-100). The residue on each screen was collected, dried at 60°C, and weighed. Sand grains with diameters less than the particle size of aggregate-associated organic matter were assumed to be a component of macro-aggregates.

### Data analysis

Soil aggregates were separated into different sizes following the method of Six *et al*. [30]. The weights of these differently sized water-stable aggregates were used to calculate parameters including mean weight diameter (*MWD*) and geometric mean diameter (*GMD*), the content of >0.25mm aggregates (*R*_*0*.*25*_), and the unstable aggregate index (*E*_*LT*_) according to the methods of Youker *et al*. as described in Hillel *et al*. [31–32]. The ratio of i-diameter aggregate weight, wet screening (*w*_*i*_) was calculated using Eq (1).

$$w_{i}=\frac{W_{w_{i}}}{50}\times 100\%$$ (1)

*R*_*0*.*25*_, *MWD*, *GMD*, and *E*_*LT*_ were computed using the dataset of differently sized aggregates:

$$R_{0.25}=\frac{M_{r>0.25}}{M_{T}}=1-\frac{M_{r<0.25}}{M_{T}}$$ (2)

$$MWD=\frac{\sum_{i=1}^{n}\bar{x_{i}}w_{i}}{\sum_{i=1}^{n}w_{i}}$$ (3)

$$GMD=Exp\left[\frac{\sum_{i=1}^{n}w_{i}{\text{ln}}^{}\bar{x_{i}}}{\sum_{i=1}^{n}w_{i}}\right]$$ (4)

$$E_{LT}=\frac{W_{T}-W_{0.25}}{W_{T}}\times 100\%$$ (5)

In the formulae, *M*_*T*_ is the sum of aggregate weight, *W*_*T*_ is the total weight of experimental soil, and *W*_*0*.*25*_ is the weight of water-stable aggregates.

Fractal dimension (*D*) was computed using the formulae derived from Jastrow *et al*. [33]:

$$\frac{M_{}\left(r<\bar{x_{i}}\right)}{M_{T}}={\left(\frac{\bar{x_{i}}}{x_{\text{max}}}\right)}^{3-D}$$ (6)

Taking logarithms of formula (6):

$$\text{lg}\left[\frac{M_{}\left(r<\bar{x_{i}}\right)}{M_{T}}\right]=\left(3-D\right)\text{lg}\left(\frac{\bar{x_{i}}}{x_{\text{max}}}\right)$$ (7)

*D* can be obtained through data fitting using formulae (6) and (7).

In the formula, $\bar{x_{i}}$ is the weight of aggregates of a certain diameter, $M_{}\left(r<\bar{x_{i}}\right)$ is the weight of aggregates of diameter less than $\bar{x_{i}}$, and ***x***_max_ is the maximum diameter of the aggregates.

Aggregate-associated C following the wet screening was determined by using potassium dichromate oxidation titration, and the aggregate-associated C storage and contributing rate were calculated using formulae (7) and (8) according to the methods of Ellert *et al*. [34].

$$\begin{array}{l} Aggreagte-associated\;C\;storage=Content\;of\;aggreagte-associated\;C\\ \times aggreagte\;content\left(\%\right)\times bulk\;density\times volume\;of\;soil\;layer \end{array}$$ (8)

$$\begin{array}{l} Contribution\;rate\;of\;aggreagte-associated\;C\;storage=Content\;of\\ aggreagte-associated\;C\times aggreagte\;content\left(\%\right)/content\;of\;SOC\;in\;raw\;soil \end{array}$$ (9)

Data processing was conducted in Microsoft Excel2003. The Least Significant Difference test (LSD) in SPSS13.0 software (SPSS Inc., Chicago, IL, USA) was employed for variance analysis and multiple comparisons (*α = 0*.*05*). Pearson’s correlation analysis was used to analyze the correlation between the soil indices. Sigmaplot12.0 was used for plotting the graphs.

## Results

### Distribution characteristics of water-stable soil aggregates

Overall, the number and size of water-stable aggregates decreased with increased soil depth from 0-60cm under all tillage treatments. Moreover, 0.25–1 and 1–2mm aggregates dominated the soil throughout the 0-60cm depth, accounting for 36.3–55.4% and 19.2–35.4% of total aggregates, with the exception of the 1-2mm aggregates in the no tillage treatment at the 50-60cm depth (Table 2). The ST treatment showed a significantly higher proportion of macro-aggregates in the top 30cm of soil than did the other treatments. For the >2mm and 1-2mm size classes, the ST treatment outperformed at least one other treatment at the 0–10, 10–20, and 20-30cm depths. For the 0.25-1mm size class, the ST treatment was significantly higher only at 0-10cm. Below 30cm, trends for macro-aggregates were less clear. Distribution of micro-aggregates in the top 20cm of soil showed the converse pattern. The MP and CT treatments had significantly higher micro-aggregate content at the 0–10 and 10-20cm depths.

**Table 2:** Water-stable aggregate contents of different classes at 0-60cm depth of soil in different tillage treatments.

Depth (cm)	Treatments	Macro-aggregate (%)			Micro-aggregate (%)			*R*_*0*.*25*_
		> 2 mm	1–2 mm	0.25–1 mm	0.053–0.25 mm	0.002–0.053 mm	< 0.002 mm	
0–10	ST	9.4±0.31a^¶^	35.1±1.93a	45.1±1.58ab	7.8±0.76b	1.5±0.13c	1.1±0.33c	89.6±0.70a
	NT	6.9±0.31b	34.7±2.13a	47.8±2.39a	7.7±0.62b	1.4±0.12c	1.4±0.24bc	89.5±0.50a
	MP	2.2±0.07c	30.6±2.22ab	37.9±2.77bc	18.2±0.25a	5.8±0.45b	5.3±0.46a	70.7±0.77b
	CT	2.3±0.18c	28.1±1.41b	36.3±3.00c	18.0±0.68a	12.2±1.63a	3.1±0.99b	66.6±1.53c
10–20	ST	9.3±1.24a	32.9±1.57a	47.0±2.62a	2.4±0.11c	2.4±0.11c	3.6±0.45a	89.1±0.67a
	NT	7.4±0.90ab	28.4±1.14ab	44.6±1.03a	3.6±0.57bc	3.6±0.57bc	3.2±1.01a	80.3±0.75b
	MP	3.7±0.18c	28.1±3.53ab	42.0±4.81a	5.4±0.13a	5.4±0.13a	4.9±1.18a	73.7±1.42c
	CT	5.5±0.46bc	24.6±0.78b	49.5±1.04a	4.4±0.55ab	4.4±0.55ab	3.0±0.56a	79.6±1.60b
20–30	ST	12.9±0.29a	30.7±2.24ab	43.5±0.61a	8.0±2.64a	4.0±1.16bc	0.8±0.40c	87.1±2.02a
	NT	6.9±0.18b	31.8±2.46a	41.6±0.64a	10.8±0.67a	5.9±0.56ab	2.7±0.54b	80.3±1.65b
	MP	9.5±1.18ab	31.5±5.92a	43.8±5.25a	9.9±1.05a	2.9±1.03c	2.3±0.24b	84.8±1.83ab
	CT	10.4±2.28ab	19.2±1.94b	42.4±0.52a	10.3±0.38a	8.2±0.37a	9.5±0.49a	72.0±0.13c
30–40	ST	9.9±0.17a	34.4±3.91a	40.4±3.22a	8.5±1.70b	4.5±1.53a	1.6±0.18a	84.8±1.02a
	NT	8.0±1.89a	32.1±4.25a	39.7±1.46a	10.7±1.24ab	6.7±2.42a	2.8±1.05a	79.8±2.29a
	MP	5.7±2.28a	29.8±2.90a	48.8±3.36a	9.4±0.42ab	4.4±1.06a	1.9±0.76a	84.3±0.96a
	CT	4.8±1.15a	21.0±11.24a	47.7±9.74a	18.0±5.24a	7.7±2.14a	0.7±0.12a	73.5±1.74b
40–50	ST	3.9±1.32a	35.4±9.74a	46.0±5.45ab	8.5±3.75a	3.9±1.37a	2.1±0.44ab	85.4±5.51a
	NT	2.0±0.58a	20.1±2.98a	58.2±5.55a	15.3±3.52a	2.0±0.57a	2.3±0.66ab	80.3±4.62a
	MP	5.3±1.48a	30.4±6.38a	43.9±3.70b	13.3±2.31a	3.3±2.44a	3.8±0.76a	79.6±3.74a
	CT	4.4±1.51a	19.7±2.27a	55.4±0.85ab	15.2±3.23a	3.7±1.25a	1.5±0.59b	79.4±3.27a
50–60	ST	2.4±1.57a	25.8±2.78a	56.3±4.77a	9.7±0.27d	2.1±0.41b	3.8±0.66a	84.8±1.40a
	NT	3.6±0.73a	15.0±0.88b	54.9±2.69a	22.1±1.08a	2.4±0.52b	2.0±0.35a	80.2±6.46a
	MP	3.1±1.08a	27.1±3.32a	44.7±4.87ab	19.3±0.41b	2.9±0.90b	3.0±0.87a	74.9±2.01ab
	CT	2.0±0.56a	27.8±1.42a	38.0±1.39b	17.0±0.26c	12.0±0.72a	3.2±0.48a	67.8±0.88b

^¶^ Data are represented as means ± S. D. and data with the same letters within each column indicate no significant difference at *P = 0*.*05*level. *R*_*0*.*25*_ is aggregates of diameter > 0.25 mm.

### Stability (*GMD*, *MWD*, *and E*_*LT*_) of water-stable soil aggregates

Stability of water-stable soil aggregates, as measured by *GMD*, *MWD*, and *E*_*LT*_ varied with soil layers under different treatments (Table 3). For the ST and NT treatments, *GMD* decreased with an increase in depth, but for the MP and CT treatments *GMD* increased initially with a subsequent decrease with depth. At the 0–10 and 10–20cm depths, ST exhibited significantly higher values of *GMD* than for the other treatments, and at each depth from 20-50cm, ST was significantly higher than at least one other treatment. There was no significant difference between the treatments at the 50–60cm depth.

**Table 3:** Distribution from 0-60cm of soil aggregate *GMD* (mm), *MWD* (mm), and *E*_*LT*_ under different tillage methods.

Parameters	Treatments	Soil depth (cm)						Average
		0–10	10–20	20–30	30–40	40–50	50–60	
*GMD*	ST	1.24±0.02a^¶^	1.21±0.06a	1.32±0.01a	1.23±0.03a	1.00±0.07a	0.85±0.06a	1.15±0.07a
	NT	1.14±0.01b	1.05±0.03b	1.06±0.02b	1.10±0.07a	0.77±0.05b	0.79±0.06a	0.99±0.07ab
	MP	0.82±0.02c	0.87±0.02c	1.19±0.10ab	1.02±0.07ab	0.99±0.01a	0.85±0.03a	0.96±0.06b
	CT	0.78±0.02c	0.94±0.02bc	1.03±0.07b	0.86±0.09b	0.86±0.07ab	0.77±0.02a	0.88±0.04b
*MWD*	ST	0.95±0.03a	0.75±0.02a	0.93±0.04a	0.86±0.08a	0.75±0.08a	0.63±0.00b	0.82±0.05a
	NT	0.89±0.02a	0.73±0.05a	0.71±0.04b	0.71±0.09a	0.69±0.02a	0.68±0.02ab	0.74±0.03a
	MP	0.56±0.01b	0.58±0.02b	0.83±0.07ab	0.74±0.03a	0.69±0.02a	0.71±0.03a	0.69±0.04ab
	CT	0.52±0.05b	0.67±0.03ab	0.40±0.02c	0.70±0.07a	0.72±0.01a	0.51±0.02c	0.59±0.05b
*E*_*LT*_	ST	10.40±0.71c	10.89±0.37c	12.86±2.01c	14.67±0.56c	14.62±5.51a	15.65±1.21c	13.19±0.88c
	NT	10.49±0.5 0c	19.70±0.75b	19.40±1.65b	20.15±0.45b	19.66±4.62a	26.45±1.26b	19.32±2.08b
	MP	29.29±0.77b	26.29±1.42a	15.16±1.83bc	15.68±0.96c	20.35±3.74a	25.11±2.01b	21.98±2.39ab
	CT	33.37±1.53a	20.43±1.61b	27.95±0.13a	26.44±1.74a	20.39±3.09a	32.18±0.88a	26.80±2.28a

^¶^ Data are represented as means ± S. D., and data with the same letters within each column indicate no significant difference at *P = 0*.*05* level.

*MWD* was higher at 0-20cm than at the 20-60cm depths for the ST and NT treatments but was opposite for the MP and CT treatments. In the top 20cm of soil, the ST and NT treatments outperformed the MP and CT treatments. The mean for the overall 0–60cm depth showed an inter-treatment comparison of ST>NT>MP>CT, with significant differences between ST/NT and CT.

*E*_*LT*_ under different tillage treatments varied with soil depth, increasing with depth for the ST and NT treatments, but initially increasing and then decreasing for the MP and CT treatments. *E*_*LT*_ was significantly higher for the CT treatment at depths of 0–10, 20–30, 30–40, and 50-60cm, and was on average higher in the CT and MP treatments.

### Influence of tillage methods on aggregate fractal dimension (*D*)

**Fig 4:**
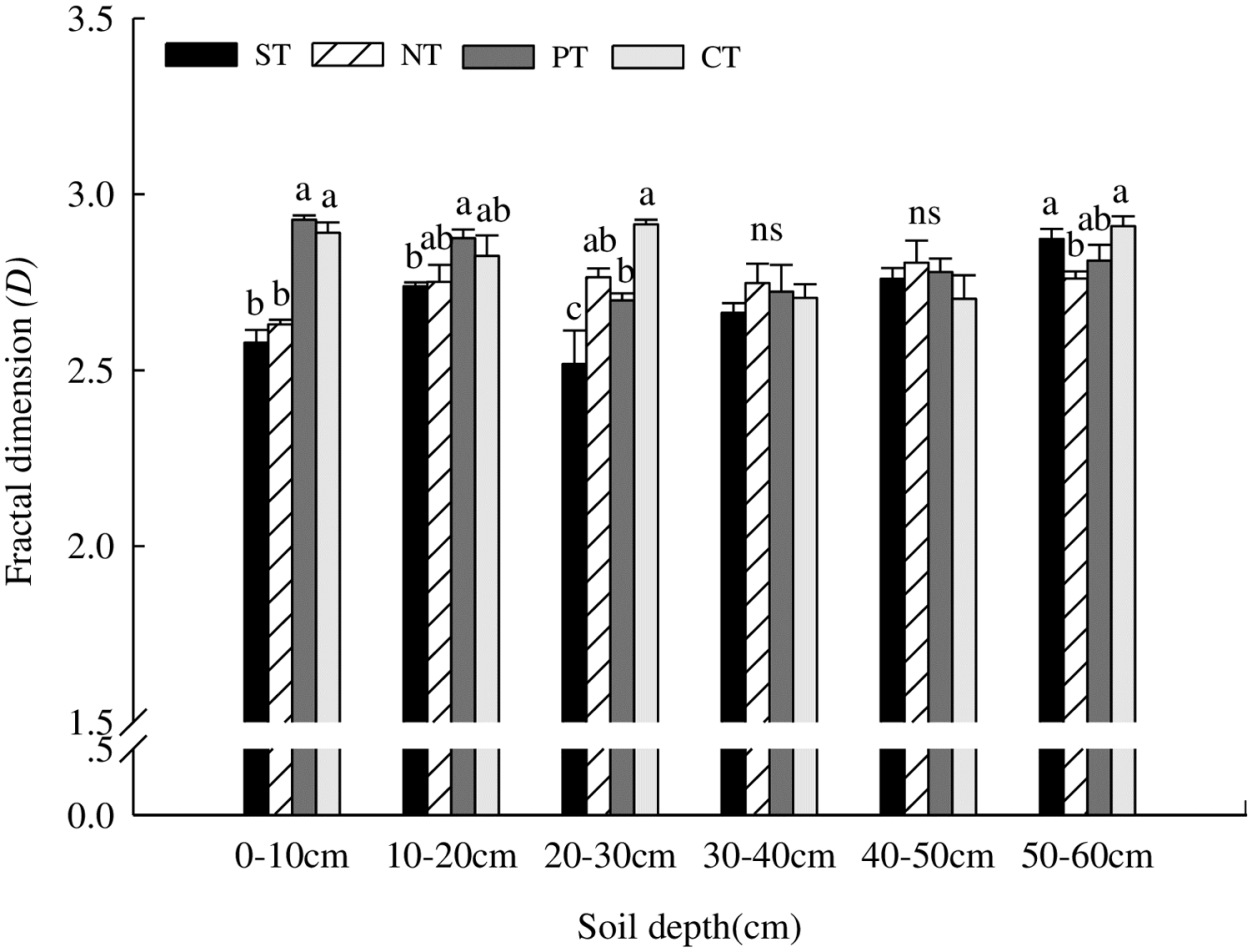
Effect of tillage methods on fractal dimension (*D*) of water-stable aggregates (*n = 3*). Lowercase letters indicate significant differences (*P≤0*.*05*) between treatments, read left to right. Error bars represent standard errors of the mean.

Aggregate *D* varied with soil depth for different treatments and was more variable in the topsoil as compared to lower soil layers (Fig 4). Aggregate *D* for the ST and NT treatments were significantly lower than for the MP and CT treatments at the 0–10cm depth. This effect for the NT treatment disappeared with increased soil depth; however, the ST treatment still showed lower *D* for the 10–20 and 20–30cm depths. This variation dwindled at lower depths until 50–60cm, where there was no significant difference in *D* between ST, NT, and MP; however, *D* was significantly lower for the CT than for the ST and NT treatments.

### Influence of tillage methods on SOC

**Fig 5:**
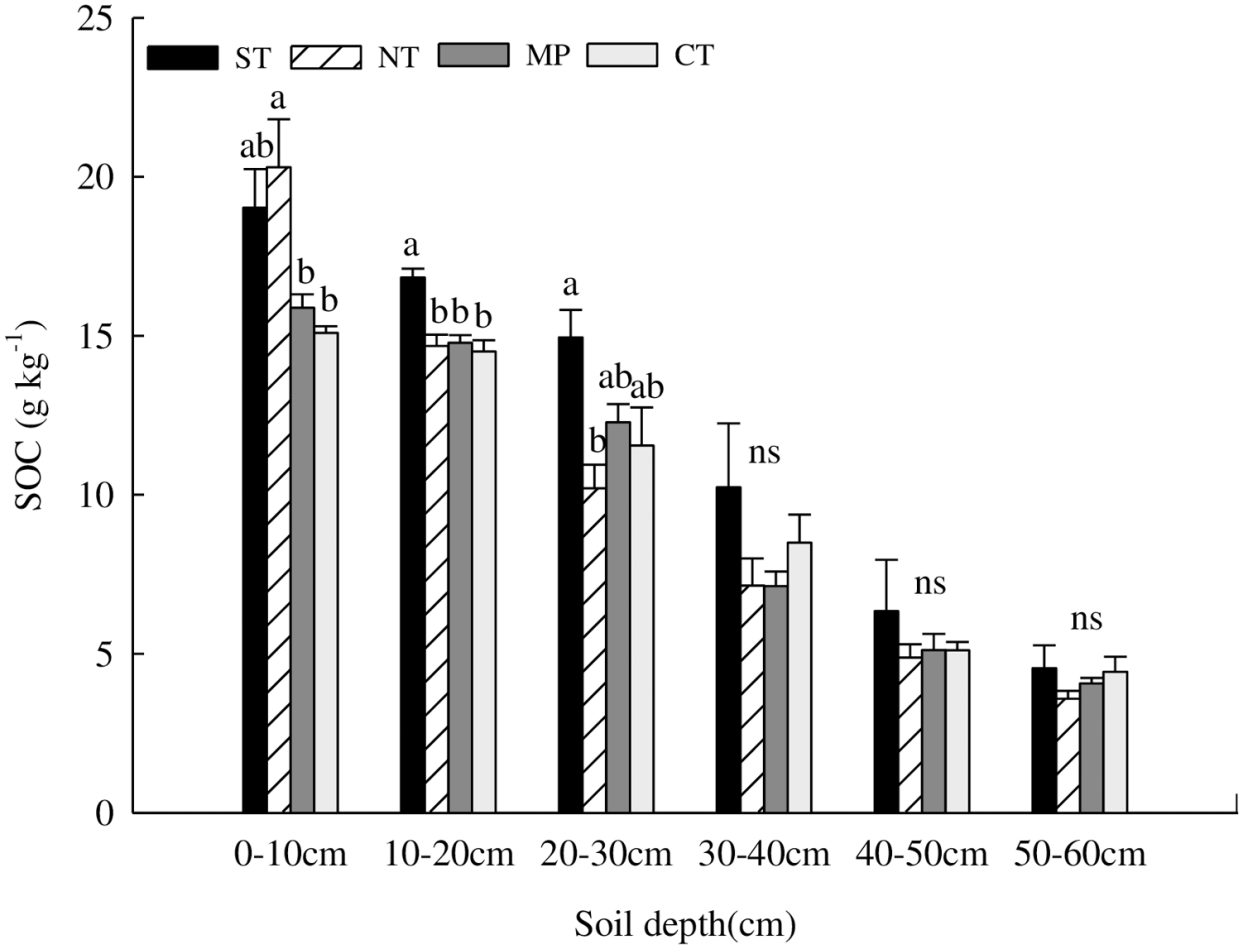
Effect of tillage methods on soil organic carbon(*n = 15*). Lowercase letters indicate significant differences (*P≤0*.*05*) between treatments. Error bars represent standard errors of the mean.

The SOC content for different treatments decreased with soil depth (Fig 5), with significantly higher content in the topsoil than in the sub-layer. At the 0–10cm depth, the mean SOC varied with treatment, with the conservation tillage (ST and NT) significantly higher than conventional tillage (CT). At 10-30cm, especially, the ST treatment was significantly higher. At 20–30cm, the mean SOC from greatest to smallest was ordered ST>MP>CT>NT, with ST significantly higher than other treatments. The mean SOC at other depths exhibited mixed patterns and no significant inter-treatment differences.

### Distribution of water-stable aggregate-associated C

Macro- and micro-aggregate-associated C decreased with an increase in the soil depth (Table 4), with a higher aggregate-associated C content in the topsoil compared to the sub-layer. Macro-aggregate-associated C content was highest in the ST treatment at the 0–10, 10–20, and 20-30cm depths for all sizes of macro-aggregates, and at the 30–40 and 40-50cm depths for macro-aggregates on average. For each depth 0-60cm, the micro-aggregate-associated C was highest in the ST treatment for water-stable aggregates of each size.

**Table 4:** Distributions of water-stable aggregate-associated C at 0-60cm depth of soil under different tillage treatments.

Depth (cm)	Treatments	Macro-aggregate-associated C (g kg^-1^)				Micro-aggregate-associated C (g kg^-1^)			
		> 2 mm	2–1 mm	1–0.25 mm	sum	0.25–0.053 mm	0.053–0.002 mm	< 0.002 mm	sum
0–10	ST	18.19±0.13a^¶^	14.54±0.05a	14.94±0.07a	47.68±0.01a	15.49±0.73a	14.96±0.03a	20.48±0.07a	50.93±0.73a
	NT	17.53±0.04b	14.58±0.01a	14.13±0.05c	46.24±0.06b	14.31±0.16ab	14.17±0.16b	15.78±0.13b	44.26±0.14b
	MP	13.98±0.05d	11.31±0.01c	13.54±0.06d	38.84±0.01 c	11.62±0.16c	11.05±0.11c	11.28±0.23d	33.95±0.04d
	CT	16.78±0.11c	13.74±0.05b	14.64±0.13b	45.16±0.07 b	13.86±0.03b	14.03±0.27b	12.22±0.01c	40.12±0.31c
10–20	ST	18.15±0.06a	15.79±0.03a	16.13±0.15a	50.08±0.21a	18.68±0.08a	16.01±0.12a	21.98±0.06a	56.67±0.14a
	NT	15.37±0.10c	14.54±0.05c	14.64±0.13b	44.55±0.28c	12.30±0.07c	13.34±0.02d	16.95±0.07c	42.60±0.12c
	MP	12.37±0.04d	13.15±0.03d	12.69±0.02c	38.21±0.10d	11.62±0.16d	13.97±0.16c	13.69±0.02d	39.28±0.01d
	CT	17.74±0.01b	15.19±0.03b	14.63±0.05b	47.56±0.08b	14.31±0.16b	15.12±0.05b	20.42±0.02b	49.84±0.14b
20–30	ST	16.22±0.16a	15.11±0.05a	15.61±0.07a	46.94±0.04a	14.49±0.01a	16.21±0.06a	18.67±0.04a	49.37±0.11a
	NT	14.01±0.05c	13.60±0.04c	13.65±0.04c	41.27±0.05c	12.76±0.09c	11.41±0.03d	13.61±0.08d	37.78±0.09d
	MP	15.48±0.09b	14.18±0.08b	14.12±0.12b	43.78±0.12b	13.96±0.08b	13.85±0.01c	17.27±0.09b	45.08±0.18b
	CT	13.79±0.09c	12.40±0.14d	12.53±0.02d	38.72±0.06d	12.54±0.01d	14.43±0.05b	15.33±0.31c	42.30±0.26c
30–40	ST	12.77±0.14b	11.07±0.15a	11.50±0.03a	35.35±0.04a	10.05±0.05a	10.04±0.07a	14.19±0.16a	34.28±0.28a
	NT	8.78±0.01c	8.25±0.04d	7.40±0.03d	24.43±0.07d	7.86±0.01c	9.21±0.15c	6.10±0.05d	23.16±0.21d
	MP	13.16±0.01a	10.47±0.11b	10.47±0.07b	34.10±0.16b	8.49±0.02b	8.39±0.04b	10.70±0.03b	27.58±0.03b
	CT	8.20±0.07d	9.01±0.03c	8.20±0.06c	25.41±0.05c	7.39±0.03d	8.08±0.01d	9.25±0.06c	24.72±0.08c
40–50	ST	11.10±0.06a	6.15±0.01b	6.62±0.01a	23.87±0.08a	6.08±0.03a	8.03±0.16a	7.92±0.12a	22.03±0.31a
	NT	7.52±0.02d	5.46±0.10d	4.58±0.10d	17.56±0.22d	4.70±0.06b	5.02±0.03c	6.10±0.05c	15.82±0.14c
	MP	10.83±0.12b	6.47±0.06a	5.06±0.03c	22.36±0.09b	4.58±0.12b	5.09±0.01c	6.03±0.02c	15.70±0.12c
	CT	8.47±0.06c	5.76±0.06c	5.80±0.05b	20.03±0.06c	5.87±0.08a	5.90±0.01b	6.91±0.10b	18.68±0.19b
50–60	ST	6.39±0.10ab	5.07±0.01b	4.06±0.03c	15.52±0.12b	5.37±0.10a	6.71±0.04a	5.60±0.05a	17.68±0.18a
	NT	6.23±0.02b	4.64±0.02c	4.29±0.06b	15.16±0.06c	3.97±0.06d	5.06±0.07c	5.55±0.01a	14.58±0.12c
	MP	6.48±0.01a	5.40±0.09c	4.72±0.01a	16.60±0.10a	4.39±0.02c	4.52±0.02d	5.15±0.05b	14.06±0.05d
	CT	4.78±0.04c	4.34±0.01d	4.12±0.03c	13.24±0.03d	4.71±0.01b	5.28±0.01b	5.54±0.06a	15.52±0.06b

^¶^ Data are represented as means ± S.D., and data with the same letters within each column indicate no significant difference at *P = 0*.*05* level.

### SOC storage in water-stable aggregates

The SOC storage in macro-aggregates under different treatments significantly decreased with soil depth (Table 5). However, no significant variation was observed in the micro-aggregate-associated C storage with depth. SOC storage increased with aggregate size from 1–2 to > 2mm and decreased with a decrease in aggregate size. The SOC storage in macro-aggregates of all sizes from 0-30cm depth was higher in the ST treatment than in other treatments. From 30-60cm, trends were less clear. SOC storage in micro-aggregates showed the opposite trend, with significantly higher levels in the CT treatment from 0-30cm, and no significant differences between treatments below this depth.

**Table 5:** Distribution of soil organic carbon storage in water-stable aggregates in different soil layers and tillage treatments.

Depth (cm)	Treatments	Macro-aggregate (t ha^-1^)				Micro-aggregate (t ha^-1^)			
		> 2 mm	2–1 mm	1–0.25 mm	Sum	0.25–0.053 mm	0.053–0.002 mm	< 0.002 mm	Sum
0–10	ST	2.65±0.74a^¶^	5.87±0.34a	7.75±0.23a	16.28±0.85a	1.38±0.11c	0.26±0.02c	0.26±0.08b	1.90±0.08c
	NT	1.40±0.07b	5.82±0.36a	7.78±0.40a	15.00±0.11a	1.26±0.10c	0.23±0.02c	0.25±0.04b	1.75±0.08c
	MP	0.35±0.01b	3.98±0.29b	5.91±0.43b	10.24±0.17b	2.44±0.06b	0.73±0.05b	0.69±0.07a	3.86±0.08b
	CT	0.44±0.04b	4.43±0.22b	6.11±0.54b	10.99±0.37b	2.88±0.08a	1.96±0.23a	0.44±0.14ab	5.28±0.20a
10–20	ST	2.43±0.03a	6.85±0.19a	9.14±0.16ab	18.42±0.29a	0.61±0.01ab	1.54±0.10c	0.72±0.01ab	2.86±0.11b
	NT	1.62±0.02b	5.04±0.25b	8.49±0.10b	15.15±0.22b	0.49±0.10b	1.40±0.03c	0.67±0.14b	2.56±0.27b
	MP	0.59±0.03d	4.02±0.31c	7.67±0.31c	12.28±0.16c	0.82±0.01a	3.27±0.06b	0.97±0.02ab	5.05±0.07a
	CT	1.35±0.09c	4.69±0.09bc	9.42±0.19a	15.46±0.36b	0.73±0.11ab	3.56±0.08a	1.05±0.17a	5.35±0.23a
20–30	ST	3.06±0.10a	6.77±0.51a	9.92±0.17a	19.75±0.47a	1.70±0.56a	0.96±0.28b	0.21±0.11c	2.87±0.44b
	NT	1.41±0.03b	6.32±0.47a	8.30±0.10ab	16.02±0.34c	1.99±0.13a	0.98±0.10b	0.54±0.11bc	3.51±0.32b
	MP	2.15±0.26b	6.52±1.23a	9.03±1.10ab	17.71±0.38b	2.03±0.22a	0.59±0.21b	0.59±0.06b	3.20±0.37b
	CT	2.09±0.46b	3.48±0.36b	7.76±0.11b	13.33±0.07d	1.88±0.07a	1.73±0.09a	2.12±0.14a	5.73±0.06a
30–40	ST	1.92±0.03a	5.74±0.61a	7.01±0.57a	14.67±0.09a	1.29±0.26a	0.68±0.24a	0.33±0.04a	2.31±0.10a
	NT	1.06±0.25ab	4.00±0.54a	4.43±0.15b	9.50±0.34b	1.27±0.15a	0.93±0.34a	0.26±0.10a	2.45±0.27a
	MP	1.12±0.45ab	4.71±0.42a	7.72±0.57a	13.56±0.23a	1.20±0.06a	0.56±0.14a	0.31±0.12a	2.07±0.12a
	CT	0.60±0.14b	2.87±1.53a	5.83±1.19ab	9.30±1.01b	2.00±0.58a	0.95±0.26a	0.10±0.02a	3.05±0.86a
40–50	ST	0.66±0.23ab	3.29±0.90a	4.60±0.55a	8.55±0.39a	0.79±0.35a	0.48±0.18a	0.26±0.06a	1.53±0.58a
	NT	0.23±0.07b	1.66±0.24a	4.02±0.36ab	5.90±0.23c	1.09±0.26a	0.16±0.04a	0.21±0.06a	1.46±0.35a
	MP	0.87±0.24a	2.97±0.60a	3.35±0.26b	7.18±0.27b	0.93±0.16a	0.25±0.19a	0.34±0.07a	1.53±0.26a
	CT	0.55±0.19ab	1.71±0.20a	4.85±0.04a	7.11±0.33b	1.35±0.29a	0.33±0.11a	0.15±0.06a	1.83±0.27a
50–60	ST	0.23±0.15a	1.99±0.21a	3.48±0.31a	5.69±0.05a	0.80±0.04b	0.22±0.04b	0.33±0.06a	1.34±0.12b
	NT	0.34±0.07a	1.06±0.06b	3.50±0.17a	4.90±0.06b	1.33±0.08a	0.19±0.04b	0.17±0.03a	1.69±0.10b
	MP	0.31±0.11a	2.21±0.25a	3.20±0.35ab	5.72±0.14a	1.29±0.03a	0.20±0.06b	0.23±0.07a	1.71±0.15b
	CT	0.15±0.03a	1.83±0.10a	2.38±0.06b	4.36±0.05c	1.21±0.02a	0.96±0.06a	0.26±0.04a	2.44±0.12a

^¶^ Data are represented as means ± S.D., and data with the same letters within each column indicate no significant difference at *P = 0*.*05* level.

### Relative contribution of SOC in water-stable aggregates of each size

The 0.25–1.00mm diameter aggregates contributed the most to SOC at the 0–10cm depth for each of the tillage treatments (Table 6). The contributing rate was 34.7%–45.7%, with that of the ST and NT treatments significantly higher than that of MP and CT. The < 0.002mm aggregates contributed the least to SOC, with a contributing rate of 1.5%–13.4%; and those of the ST and NT treatments were significantly lower than those of MP and CT. The total contributing rate of SOC at all depths in macro-aggregates was in the order NT>ST>CT>MP, while that for micro-aggregates was MP>ST>CT>NT.

**Table 6:** Contributing rates of SOC in water-stable aggregates in different soil layers for different tillage methods.

Depth (cm)	Treatments	Macro-aggregate (%)				Micro-aggregate (%)			
		> 2 mm	2–1 mm	1–0.25 mm	Sum	0.25–0.053 mm	0.053–0.002 mm	< 0.002 mm	Sum
0–10	ST	11.5±0.30a^¶^	34.5±2.02a	42.9±1.88ab	88.9±0.46a	8.1±0.65c	1.5±0.13b	1.5±0.46c	11.1±0.47c
	NT	9.9±0.25b	34.2±2.13a	45.7±2.37a	89.7±0.47a	7.4±0.60c	1.4±0.13b	1.5±0.26c	10.3±0.47c
	MP	2.0±0.06c	23.4±1.70b	34.7±2.55c	60.1±1.03c	14.3±0.35b	12.1±1.32a	13.4±0.26a	39.9±1.02a
	CT	2.6±0.21c	26.1±1.30b	35.6±2.93bc	64.2±1.91b	16.9±0.49a	11.5±1.33a	7.4±2.78b	35.8±1.91b
10–20	ST	12.2±1.65a	31.1±1.64a	41.2±0.82ab	84.5±0.73a	3.3±0.13c	8.4±1.01b	3.8±0.15c	15.5±0.73b
	NT	8.2±0.99b	31.6±0.58a	43.6±1.41a	83.4±0.99a	3.8±0.14bc	7.6±0.65b	5.2±0.20b	16.6±0.99b
	MP	3.5±0.19c	28.5±2.24a	36.9±3.22bc	68.9±1.18b	6.5±1.02a	18.7±0.37a	5.8±0.52b	31.1±1.18a
	CT	6.5±0.11bc	26.7±1.73a	34.7±0.26c	67.9±0.64b	5.3±0.29ab	19.3±0.08a	7.51±0.41a	32.07±0.64a
20–30	ST	13.4±2.81a	30.3±1.29a	40.6±0.99a	84.3±2.38a	9.3±3.06a	5.2±1.51b	1.2±0.60c	15.7±2.38b
	NT	7.7±0.19a	34.5±2.58a	38.6±1.33a	80.8±1.74a	10.9±0.69a	5.4±0.53b	3.0±0.60bc	19.2±1.74b
	MP	11.8±1.44a	31.0±2.21a	39.8±1.61a	82.5±2.03a	11.1±1.19a	3.2±1.15b	3.2±0.33b	17.5±2.04b
	CT	11.4±2.52a	19.0±1.95b	38.2±0.69a	68.7±0.35b	10.3±0.39a	9.5±0.47a	11.6±0.76a	31.3±0.35a
30–40	ST	15.1±0.09a	26.6±0.68ab	39.8±0.31a	81.5±0.82ab	8.0±0.76b	7.6±1.19ab	2.9±0.38ab	18.5±0.82bc
	NT	10.6±1.73b	28.3±1.29ab	35.9±0.80a	74.8±2.22b	8.8±1.00b	10.3±0.70a	6.1±1.93a	25.2±2.22b
	MP	11.3±1.81ab	35.6±3.45a	35.7±2.95a	82.6±0.82a	10.1±0.19b	5.6±0.13b	1.8±0.49b	17.4±0.81c
	CT	6.1±0.48c	15.6±6.89b	43.1±10.69a	64.8±3.32c	21.3±2.49a	9.9±1.11a	3.9±0.28ab	35.2±3.32a
40–50	ST	5.2±1.34b	5.9±2.09ab	3.4±0.77ab	85.5±3.92a	5.2±1.34b	5.9±2.09ab	3.7±0.77ab	14.5±3.92b
	NT	13.6±3.26a	8.2±1.03a	5.9±1.74a	72.3±3.22b	13.6±3.26a	8.2±1.03a	5.9±1.74a	27.7±3.22a
	MP	10.4±1.20ab	0.7±0.36c	4.8±0.44ab	84.2±0.41a	10.4±1.20ab	0.7±0.36c	4.8±0.44ab	15.9±0.40b
	CT	13.8±1.25a	3.4±1.58bc	2.3±0.90b	80.5±0.58ab	13.8±1.25a	3.4±1.58bc	2.3±0.90b	19.5±0.58ab
50–60	ST	7.1±1.40a	24.0±3.65a	39.8±2.88a	70.9±2.51a	10.9±2.88a	8.9±1.30b	9.4±1.03b	29.1±2.51b
	NT	6.3±2.06a	16.4±3.29a	37.5±1.85a	60.1±1.51b	14.3±1.85a	12.5±0.90a	13.1±0.85a	39.9±1.52a
	MP	6.7±4.21a	24.4±2.03a	34.3±3.70a	65.3±1.66ab	13.8±3.70a	10.3±0.35a	10.5±0.99ab	34.7±1.66ab
	CT	6.1±2.72a	19.6±1.03a	34.3±1.03a	59.9±1.70b	13.0±1.03a	13.1±0.20ab	13.9±1.34a	40.1±1.70a

^¶^ Data are represented as means ± S.D., and data with the same letters within each column indicate no significant difference at *P = 0*.*05* level.

### Correlation between SOC, water-stable aggregates, and structure stability

Overall, there was a significant positive correlation between aggregate-associated C and SOC (*P*<0.01; Table 7). The 0.002–0.053, 0.053–0.25, 1–2 and >2mm aggregate-associated C showed significant and positive correlations with <0.02mm aggregate-associated C (*P*<0.01). There was a significant and positive correlation between >0.25mm aggregates and *E*_*LT*_, *MWD* exhibited a significant and positive correlation with 0.002–0.053, 0.25–1, and 1–2mm aggregate-associated C, but was negatively correlated with *D*. There was a significant negative correlation between *GWD* and *D*. There were no other significant correlations between other indices.

**Table 7:** Correlation between the stability parameters of aggregates, aggregate-associated C and total SOC in 0–60 cm depth soils.

	SOC	> 0.25 mm water-stable aggregates	*MWD*	*GMD*	*E*_*LT*_	*D*	> 2 mm aggregate-associated C	1–2 mm aggregate-associated C	0.25–1.00 mm aggregate-associated C	0.053–0.250 mm aggregate-associated C	0.02–0.053 mm aggregate-associated C
> 0.25 mm water-stable aggregates	0.35										
*MWD*	0.57	0.71									
*GMD*	0.36	0.73	0.64								
*E*_*LT*_	-0.34	-1.00**	-0.72	-0.74							
*D*	-0.21	-0.75	-0.79*	-0.91**	0.76*						
> 2 mm aggregate-associated C	0.96**	0.57	0.71	0.47	-0.56	-0.37					
1–2 mm aggregate-associated C	0.93**	0.51	0.79*	0.39	-0.50	-0.36	0.96**				
0.25–1.00 mm aggregate-associated C	0.95**	0.49	0.78*	0.41	-0.48	-0.37	0.97**	1.00**			
0.053–0.25 mm aggregate-associated C	0.96**	0.44	0.74	0.34	-0.43	-0.30	0.97**	0.99**	1.00**		
0.02–0.053 mm aggregate-associated C	0.94**	0.46	0.76*	0.32	-0.45	-0.30	0.96**	1.00**	0.99**	1.00**	
< 0.02 mm aggregate-associated C	0.91**	0.49	0.75	0.28	-0.48	-0.27	0.95**	0.99**	0.98**	0.98**	0.99**

* and ** in the table express a significant level at *P<0*.*05* and *P<0*.*01*, respectively.

## Discussion

### Distribution and stability of water-stable aggregates

Soil aggregates are the foundation of the soil structure and soil substance, energy conservation, and metabolism [3–4]. The quantity and quality of soil aggregates directly determine soil quality and fertility [1–2, 17]. The stability of soil aggregates determines the ability of the aggregates to resist exogenic action and to remain stable when exposed to changes in the external environment. In addition, aggregates are known to closely correlate with the soil erodibility and appear to play an important role in maintaining the stability of soil structure. Our results showed that the conservation strategies of spacing tillage and no-tillage improved soil structure and increased the number of macro-aggregates by reducing the disturbance frequency of tillage and keeping high stubble cover, which served to prevent erosion. This result is in agreement with previous findings from long-term studies in geographic areas across similar latitudes [35].

Using suitable tillage and increasing the soil organic matter can improve the formation of soil aggregates and increase their stability [36]. We found that the no-tilling (NT) method promotes the formation of soil aggregates in the topsoil (0-10cm depth) and improves the aggregate stability due to the presence of high stubble. However, the MP and CT treatments strongly disturb the soil, which can reduce the aggregate degree and stability of soil aggregates at the tillage depth of 0-20cm due to erosion and rainfall. Another demonstrated advantage of deep tillage was the 34.49% increase in the number of water-stable aggregates under the ST treatment compared to the other treatments, which could improve the formation of soil aggregate structure in the black soil of Northeastern China. Furthermore, spacing tillage (ST) promoted the enrichment of > 0.25mm water-stable aggregates, thereby improving the soil structure. Our study showed a greater influence of tillage treatment on macro- and micro-aggregates at 0–10, 10–20, and 20–30cm layers than at other depths, suggesting an aggregate stratification phenomenon. This is due to the result of different operations of the secondary tillage. An additional reason may be the difference in the straw returned to soil under the different tillage systems.

Moldboard plowing is considered to be one of the main factors resulting in the decline of soil aggregate quality on clay loam soils in New York [37]. In contrast, conservation tillage (NT and ST) can reduce soil erosion, increase the abundance of water-stable macro-aggregates and improve their structural stability, and improve the soil structure [21]. Our study suggests that *GMD* and *MWD* decreased with an increase in the soil depth and exhibited higher values for the ST and NT treatments than for the MP and CT treatments. The NT treatment showed the greatest effect at the 0–10cm depth, while the ST treatment had the greatest effect at 10–30cm. However, *E*_*LT*_ of ST and NT showed the opposite trend, when compared with the MP and CT treatments. In our study, the ST and NT treatments effectively controlled soil erosion by retaining straw on the soil surface, where the decomposed straw can promote the formation of particulate organic matter inside micro-aggregates that further increase soil structure stability. Our study also found minimal *D* (aggregate fractal dimension) value at the 0–10cm depth for the ST and NT treatments, mainly due to straw return to the field, and to the decline in disturbance promoting aggregate formation [36]. However, the effect of NT gradually disappeared with increasing soil depth, although ST still showed higher values at 10–20 and 20–30cm. This was largely due to the appropriate soil environment created by the sub-soiling effect, with a positive effect on the formation of the soil aggregates [38]. In addition, *D* responded rapidly to the long-term influence of tillage treatments and can be used as an indicator of soil aggregate stability [9]. The 0.25–1.00 and 1–2mm aggregates dominated all soil depths by responding quickly to the tillage treatments and thus can also be used as an index to assess the long-term influence of the tillage treatments on aggregate characteristics. Our results were consistent with those of Liang *et al*. [39].

### SOC and aggregate-associated C

SOC is an important index of soil quality and health and is an important component of the soil fertility of farmlands, as well as being the core of soil quality and function [4]. SOC content can directly affect soil fertility and crop yield, and greatly affects the formation and stability of the water-stable soil aggregate structure [40]. Our study showed that SOC decreases with soil depth and is more abundant in topsoil (0-20cm) than in the sub-layers (below 20cm). SOC content was highest at the 0–10 and 10–20cm depths, accounting for 27.98 and 24.28% of the total SOC, respectively. NT showed a high SOC accumulation at 0–10cm, while ST promoted accumulation at all depths 0–60cm, with significantly higher accumulation at 0–10, 10–20 and 20–30cm, which was consistent with the results found by Zhang *et al*. [27]. The mechanism and reason for this phenomenon were the higher straw cover in the NT and ST treatments, which can reduce soil erosion, land surface evaporation, and loss of soil organic matter, and can improve the soil structure. Another reason is that straw mulching has a moisture preservation effect and benefits the activities of microorganisms, which can accelerate the SOC turnover. Furthermore, the subsoiling effect can promote root system growth, and a large amount of the root system and stubble can be converted into SOC through decomposition and humification effects, thereby increasing the SOC content in deep soil and enhancing the soil ability to accumulate C under the ST treatment. The advantage was dependent on tillage depth and healthy soil structure throughout the soil profile. Varsa *et al*. reported similar results from a study on soils in Southern Illinois conducted during 1989–1993 [41].

Soil structure and the interactions between aggregates determine the quality of the SOC pool [1]. Stable soil structure protects the soil organic matter from rapid decomposition [8]. A previous study showed that SOC content increases with an increase in aggregate size [11]. Our study suggests that the SOC content of aggregates decreased with the deeper soil layers within 0–60cm for the different tillage treatments, which was consistent with the results of Zheng *et al*. [42]. The aggregate-associated C in the NT and ST treatments were higher than for the MP and CT treatment sat the 0–10cm depth due to the amounts of organic matter and favorable soil structure. However, the aggregate-associated C of the NT treatment decreased with soil depth but remained high under the ST treatment. Our study concluded that conservation tillage can significantly increase the SOC content in soil, and that this effect is significantly stronger in ST and NT than in MP and CT at 0-60cm depths. Although the highest SOC content was observed in the 0.25–2.00mm aggregates, mass fraction for this size class was relatively low. Thus, our study concluded that the increase in SOC was mainly due to the increase in SOC in >2mm water-stable aggregates [24].

### Soil aggregate-associated C storage and contributing rate

Soil aggregates have three major effects on soil [15]. They regulate and maintain water, fertilizer, gas, and heat in the soil, affect the types and activity of the soil enzymes, and also maintain and stabilize the loose arable layer [35]. Almost 90% of SOC exists in the form of aggregates in the topsoil. Therefore, study of intra-aggregate C is of great significance to the influence of human disturbance on SOC [42]. In our study, the ST and NT treatments not only increased the content of the water-stable aggregates, but also increased the SOC content, similar to the observations made by Tisdal *et al*. [43]. The sum of contributing rates of macro-aggregates was superior to that of the micro-aggregates. The highest SOC storage was for 0.25–1and 1–2mm aggregates because more SOC accumulation occurred in those aggregates. There were more macro-aggregates in the ST and NT treatments than in the MP and CT treatments, which showed more micro-aggregates, with their turnover closely related to SOC storage. Protection and maintenance of the macro-aggregate stability and ratio are of great importance in the sustainability of soil fertility [25]. In addition, the contributing rate of SOC in differently sized aggregates decreased, consistent with the trend of soil aggregate-associated C storage and SOC with increasing soil depth.

In this study, the contributing rate of water-stable aggregates to SOC showed mixed patterns with soil depth; however, the contributing rate of SOC was higher for the ST and NT treatments than for the MP and CT treatments, suggesting that over-frequent tillage can accelerate the destruction of newly formed macro-aggregates, thus reducing aggregate stability. Conversely, the NT treatment can compact the topsoil, preventing formation of macro-aggregate structures. The ST treatment could be a supplement for the NT treatment through a sub-soiling effect, which could promote macro-aggregate formation and stability by improving soil structure and the contributing rate of the macro-aggregate-associated C in deep layer soil [44]. In addition, there was a significant correlation between SOC and aggregate-associated C of various sizes (correlation coefficients of 0.91–0.96), suggesting that aggregate-associated C made an important contribution to SOC accumulation and enrichment and played a vital role in the soil C pool balance (Table 2).

## Conclusions

Our study suggests that different tillage treatments affected the water-stable soil aggregate distribution in the black soil in Northeast China. The conservation tillage (ST and NT) treatments effectively improved the soil structure and strengthened the stability of water-stable soil aggregates. In addition, they increased the SOC content and storage in aggregates of different sizes with comparison of MP and CT. Furthermore, long-term adoption of conservation tillage methods significantly increased the content of water-stable macro-aggregates and of aggregate *MWD*, and increased the SOC content, ratio of, and storage in the macro-aggregates. In particular, the ST treatment increased the SOC content and enriched the newly formed C in macro-aggregates.

In addition, correlation analysis suggested a significant correlation between SOC and aggregate-associated C in differently sized aggregates. The 0.25–1 and 1–2mm aggregates were the main sites of SOC storage and were also the important indices of the soil C pool saturation. The relative abundance of 0.25–1 and 1–2mm aggregates could be used as indicators for the long-term influence of different tillage treatments on aggregate characteristics and *D* can also be a good index of the soil aggregate stability.

## Supporting information

S1 File Data for the cited Figs 2, 4 and 5 in the manuscript. (XLSX)

S2 File Data for the cited Tables 2–7 in the manuscript. (XLSX)
